# Efficient One-Pot Synthesis of a Hexamethylenetetramine-Doped Cu-BDC Metal-Organic Framework with Enhanced CO_2_ Adsorption

**DOI:** 10.3390/nano9081063

**Published:** 2019-07-24

**Authors:** Aisha Asghar, Naseem Iqbal, Tayyaba Noor, Majid Ali, Timothy L. Easun

**Affiliations:** 1 U.S Pakistan Centre for Advanced Studies in Energy, National University of Sciences and Technology, H-12, Islamabad 44000, Pakistan; 2 School of Chemical and Materials Engineering, National University of Sciences and Technology, H-12, Islamabad 44000, Pakistan; 3 School of Chemistry, Cardiff University, Main Building, Park Place, Cardiff CF10 3AT, UK

**Keywords:** energy efficiency, functional metal organic frameworks, Cu-BDC, HMTA, CO_2_ adsorption

## Abstract

Herein we report a facile, efficient, low cost, and easily scalable route for an amine-functionalized MOF (metal organic framework) synthesis. Cu-BDC⊃HMTA (HMTA = hexamethylenetetramine) has high nitrogen content and improved thermal stability when compared with the previously reported and well-studied parent Cu-BDC MOF (BDC = 1,4-benzenedicarboxylate). Cu-BDC⊃HMTA was obtained via the same synthetic method, but with the addition of HMTA in a single step synthesis. Thermogravimetric studies reveal that Cu-BDC⊃HMTA is more thermally stable than Cu-BDC MOF. Cu-BDC⊃HMTA exhibited a CO_2_ uptake of 21.2 wt % at 273 K and 1 bar, which compares favorably to other nitrogen-containing MOF materials.

## 1. Introduction

The world is currently facing the urgent and demanding challenges of saving and utilizing energy as efficiently as possible. Ever increasing carbon dioxide levels in the atmosphere are a serious threat to the environment [1]. Various carbon capture techniques have been explored to mitigate carbon dioxide levels in the atmosphere, including point source CO_2_ capture using advanced materials [2]. Metal−organic frameworks (MOFs) are an advanced class of microporous and often crystalline nanomaterials comprised of metal coordination sites bridged by organic linkers [3,4]. The resulting organic/inorganic hybrid 3-D networks that form often contain well-defined porosity, high surface area, and tunable chemical functionalities with potential for versatile applications in catalysis [5,6], separations [7], and gas storage [8]. With respect to the last of these, many studies have investigated the capture of carbon dioxide gas. Amine sites have an affinity towards carbon dioxide, are known to be highly effective at enhancing CO_2_ adsorption, and are amenable to use under dry or humid conditions. [9]. In this paper, we describe the synthesis, characterization and CO_2_ sorption of a hexamethylenetetramine-doped metal-organic framework. This study is an effort to incorporate hexamethylenetetramine within a Cu-BDC (BDC = 1,4-benzenedicarboxylate) framework, using an in-situ modification during synthesis, and to study the effect on carbon dioxide gas sorption capacity. Herein, we report a very straightforward method for modification of already reported Cu-BDC [10]. The strategy has several advantages. First, the entire synthetic procedure is quite simple. Second, this method is efficient, with the potential for high-yields, and hexamethylenetetramine is a low-cost chemical (£15.68/kg [11]). Third, a high nitrogen content can be achieved in the resulting Cu-BDC⊃HMTA material.

## 2. Materials and Methods

All the chemicals were purchased from Sigma Aldrich/Merck (St. Louis, MO, USA) and used as received.

### 2.1. Synthesis

**Figure 1:**
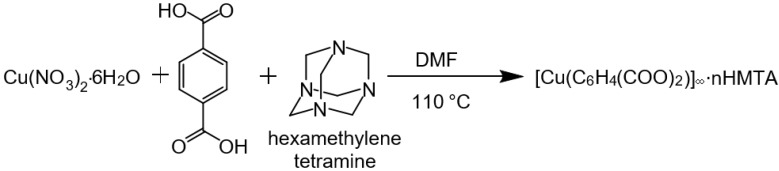
Reaction scheme for Cu-BDC⊃HMTA synthesis.

To prepare Cu-BDC⊃HMTA, equimolar quantities (1:1:1) of Cu(NO_3_)_2_·6H_2_O (296 mg, 1 mmol), terephthalic acid (166 mg, 1 mmol) and hexamethylenetetramine (140 mg, 1 mmol) were dissolved in 10 mL DMF in a 50 mL beaker. The contents were ultrasonicated at 25 °C for 30 min, and then the solution was transferred to a 23 mL Teflon vial in a steel Parr vessel. The Parr vessel was sealed and heated in an oven at 110 °C for 24 h to yield greenish-blue crystals. The reaction mixture was decanted, the product washed three times with DMF (5 mL), and then three times with THF (5 mL). This yielded blue crystals. The sample was activated in a vacuum oven at 130 °C for 12 h before further analysis. The same synthesis strategy was used for obtaining Cu-BDC MOF, without the addition of HMTA [10]. This yielded blue crystals. A schematic reaction scheme for Cu-BDC⊃HMTA synthesis is shown in Figure 1. 

### 2.2. Characterization 

Powder X-ray diffraction (PXRD) patterns were collected on an X’PertPro Panalytical Chiller 59 diffractometer (Malvern, UK) using copper K*α* (1.54 Å) radiation. Diffraction patterns were recorded in the 2θ range, from 4.00 to 39.09 degrees, with a 2θ step size of 0.017, and a scan per step of 34.9 s. Elemental analyses (N, C and H) of prepared Cu-BDC⊃HMTA samples were performed to confirm presence of amine in the prepared material, using a FlashSmart NC ORG elemental analyzer (Oxford, UK). Thermogravimetic analyses (TGA) were performed using a Perkin Elmer Pyris 1 thermo-gravimetric analyzer (Champaign, IL, USA). The temperature was increased from 25 °C to 700 °C at a heating rate of 5 °C min^−1^ under a flow of air (20 mL min^−1^). SEM images were collected using TESCAN/VEGA-3 equipment (Brno, Czech Republic). A SHIMADZU IR Affinitt-1S spectrometer (Kyoto, Japan) was used to obtain IR spectra.

Prior to CO_2_ sorption studies, the samples were degassed at 130 °C for 10 h and then back-filled with helium gas. CO_2_ adsorption experiments were performed on a Quantachrome Isorb-HP100 volumetric type sorption analyzer (Boynton Beach, FL, USA). The sample was tested for adsorption at two different temperatures: 0 °C and 25 °C, at pressures from 1–14 bar. N_2_ adsorption studies of the MOF were conducted to analyze surface area and pore volume using a Quantachrome Nova 2200e at −196 °C at a relative pressure of P/P⁰ = 0.1–1.0 and prior to the measurement, samples were degassed at 160 °C under vacuum for 11 h. 

## 3. Results and Discussion

### 3.1. PXRD Patterns of Cu-BDC and Cu-BDC⊃HMTA

**Figure 2:**
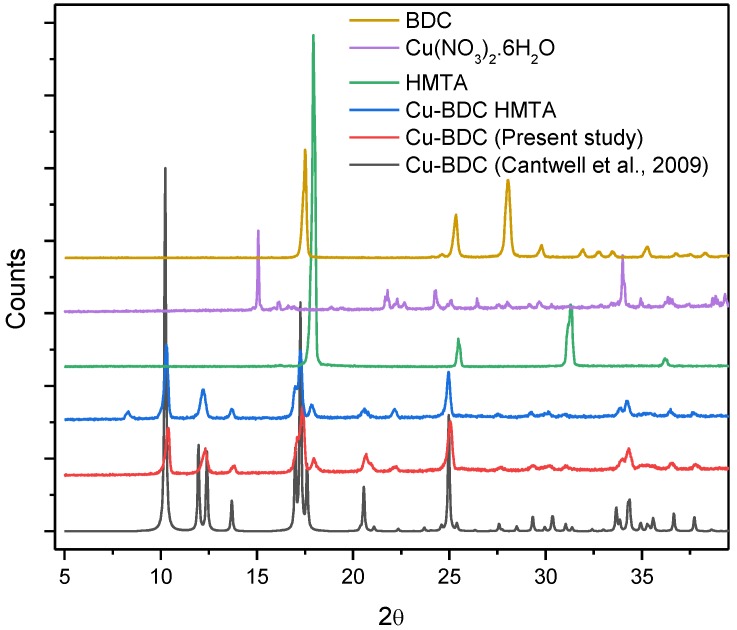
PXRD patterns for Cu-BDC (black, reported by Carson et al., 2009), Cu-BDC (red, synthesized herein), Cu-BDC⊃HMTA (blue), HMTA (green), Cu(NO_3_)_2_·6H_2_O (lilac) and the H_2_BDC linker (dark yellow).

Powder X-ray diffraction patterns, for products of the synthesis of Cu-BDC and Cu-BDC⊃HMTA, were collected and are shown in Figure 2. Both synthesized materials show sharp diffraction peaks indicating predominantly crystalline material. The peak positions are in good agreement with the PXRD of Cu-BDC previously reported by Carson et al. in 2009, indicating successful synthesis of the Cu-BDC [10]. There are several additional peaks in the PXRD pattern of Cu-BDC⊃HMTA, most notably at 2θ = 8.31°. These cannot be ascribed to a simple mechanical mixture of Cu-BDC and HMTA as, firstly, HMTA is soluble in the organic solvents used to wash the reaction product, which makes this option unlikely. Secondly, the increased CO_2_ sorption observed in the Cu-BDC⊃HMTA product (described below) is not consistent with a simple physical mixture of Cu-BDC and HMTA, which we would anticipate having a lower gas uptake than the pure Cu-BDC alone. Thirdly, and most conclusively, the powder XRD pattern of pure HMTA is shown in Figure 2, and no HMTA peaks correspond to the new peaks observed in Cu-BDC⊃HMTA. We tentatively ascribe the additional peaks to well-ordered HMTA molecules binding to the axial copper sites in the framework. Inspecting the (previously reported) Cu-BDC crystal structure [10] shows that the 2D layers pack with significantly offset paddlewheels from one layer to the next, with copper centers separated by 6.331 Å (Cu–Cu distance). Looking approximately along the Cu–Cu axis, although still with some notable offset, the next available paddlewheel is 9.068 Å (Cu–Cu distance) away. HMTA has four potentially coordinating nitrogen atoms separated by ~2.47 Å (N–N) across a tricyclic, adamantane-like cage [12]. There are examples of HMTA bridging copper nodes in a metal-organic framework, but these typically involve much shorter Cu–Cu distances (5.761 Å in the cited example) with an angle between the Cu-N bonds of adjacent centres across the HMTA of ~108°, which is not possible in our Cu-BDC⊃HMTA framework [12]. Further indirect evidence for bonding of HMTA to the framework is that it is not readily removed by simple washing with organic solvents. It has not been possible at this stage to synthesize crystals of Cu-BDC⊃HMTA of sufficient size and quality to obtain the single-crystal X-ray structure.

### 3.2. FTIR of Cu-BDC and Cu-BDC⊃HMTA

**Figure 3:**
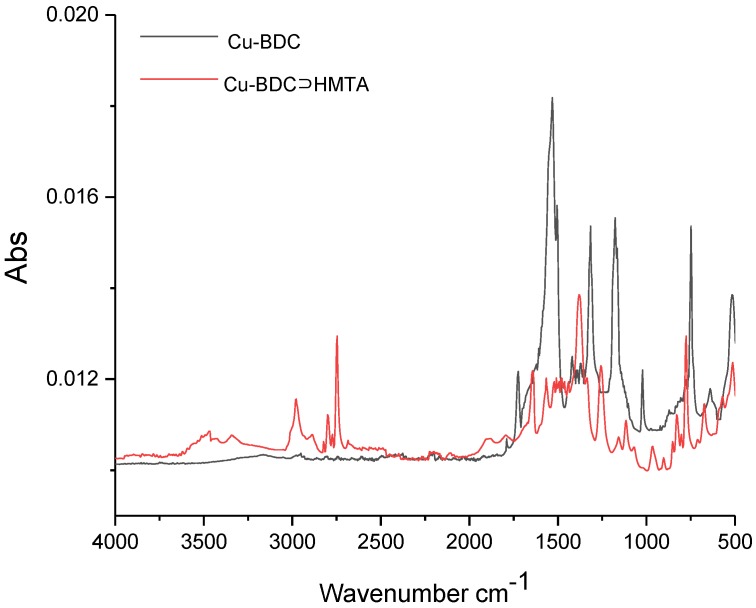
FTIR spectra for Cu-BDC and Cu-BDC⊃HMTA.

Fourier transform infrared spectra (FTIR) collected for prepared materials confirm the presence of representative functional groups indicative of Cu-BDC MOF formation (Figure 3). Sharp peaks representative of symmetric and asymmetric stretching of carboxylates bonded to Cu are observed at 1521 cm^−1^ and 1362 cm^−1^ in the Cu-BDC sample [10]. Both materials show the presence of what is likely to be water (even after vacuum-oven drying the samples) in the form of a broad peak centered around 3400 cm^−1^, which is much more evident in the Cu-BDC sample than in the Cu-BDC⊃HMTA material and is likely due to the rapid uptake of atmospheric water when performing the measurement in air. The relatively reduced water content in the Cu-BDC⊃HMTA sample may indicate slower water adsorption as a result of pore-blocking by adsorbed HMTA and, in agreement with the binding of HMTA to copper nodes proposed above, the occupation of axial sites on the copper paddlewheels by HMTA which otherwise could rapidly adsorb water. In addition to peaks coincident with those of Cu-BDC MOF, the Cu-BDC⊃HMTA sample illustrates some new features. A characteristic peak for amine-containing functional groups is observed at 1089 cm^−1^, consistent with C–N bond stretching [13,14]. Peaks at 2915 and 2845 cm^−1^ can be ascribed to stretching vibrations of C–H bonds introduced by the incorporation of HMTA [14,15].

### 3.3. Thermal Stability of Cu-BDC and Cu-BDC⊃HMTA

**Figure 4:**
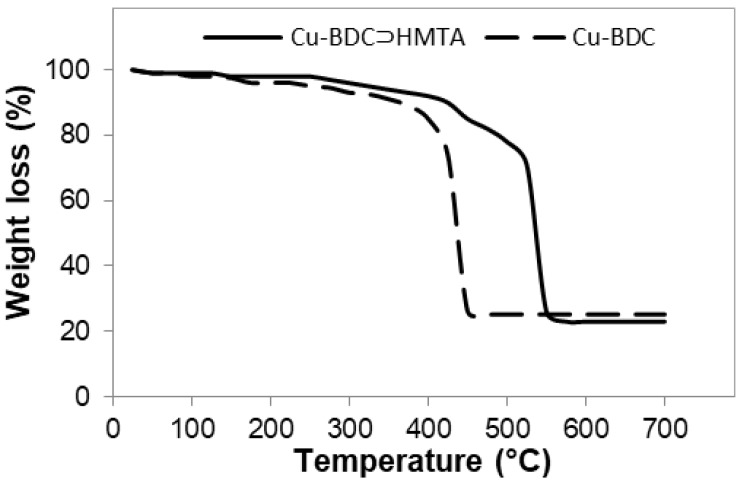
TGA of Cu-BDC MOF (dashed line) and Cu-BDC⊃HMTA (solid line).

The two materials were studied by TGA and the results are shown in Figure 4. For both MOFs there is less than 2% weight loss observed below 150 °C, indicating the pre-treatment has removed the majority of the residual solvent, and there is only minimal adsorbed moisture. The small weight loss in Cu-BDC between 170 °C and 320 °C (approx. 8%) is consistent with loss of surface adsorbed DMF [13]. For Cu-BDC, decomposition of the benzene dicarboxylate starts at about 375 °C, above which temperature there is rapid degradation to the metal oxide. Notably, there is multi-step degradation of Cu-BDC⊃HMTA, with mass losses from ~250 °C and 425 °C consistent with HMTA sublimation and thermal degradation, respectively. In Cu-BDC⊃HMTA, linker degradation appears to be overlapped with HMTA degradation. No further weight loss was observed above 450 °C for Cu-BDC MOF, and above 550 °C for Cu-BDC⊃HMTA. The Cu-BDC thermal degradation results in 25% metal oxide, and 74% linker + DMF in the initial mass, consistent with the expected metal:linker:DMF ratio of 1:1:1. The relative proportions of Cu-BDC⊃HMTA components cannot reliably be extracted from these data due to the difficulty disentangling the overlapping mass losses of HMTA and linker in this experiment.

### 3.4. Elemental Composition of Cu-BDC and Cu-BDC⊃HMTA

To confirm the chemical composition of both samples, elemental analysis and EDS were performed (Table 1). The empirical formulae calculated on the basis of EDS, and elemental analysis for Cu-BDC, and Cu-BDC⊃HMTA are: C_11_H_11_CuNO_5_ and C_14_H_16_CuN_4_O_4,_ respectively. This is consistent with metal: linker: {DMF or HMTA, respectively} molar ratios of 1:1:1, and in line with each of the copper axial sites being occupied by HMTA in Cu-BDC⊃HMTA.

### 3.5. Morphology of Cu-BDC and Cu-BDC⊃HMTA

**Figure 5:**
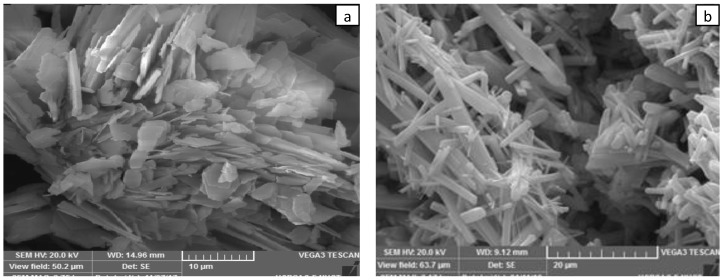
SEM images at 10 µm for (**a**) Cu-BDC MOF and at 20 µm for (**b**) Cu-BDC⊃HMTA MOF.

Scanning electron microscopy images of the prepared samples are shown in Figure 5. The Cu-BDC crystallites have a plate like structure, while Cu-BDC⊃HMTA shows a more regular flat rod-like structure.

### 3.6. CO_2_ Adsorption Studies of Cu-BDC MOF and Cu-BDC⊃HMTA

**Figure 6:**
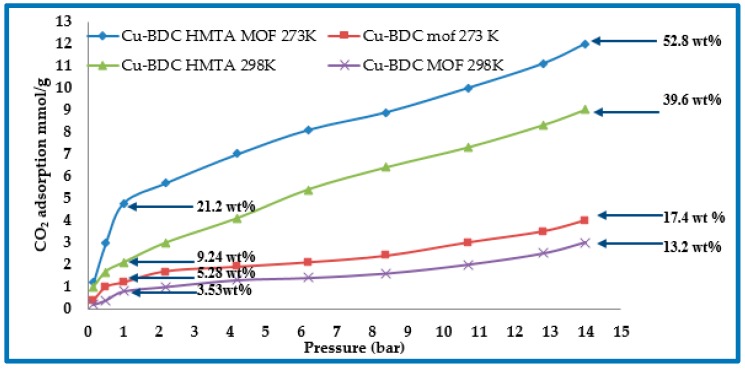
CO_2_ adsorption isotherms for Cu-BDC and Cu-BDC⊃HMTA at 273 K and 298 K, as shown.

The CO_2_ adsorption capacity for both MOF materials was evaluated by monitoring pseudo equilibrium adsorption uptake. Samples were initially degassed at 130 °C for 12 h. 200 mg of each sample was used for three consecutive adsorption-desorption cycles at 273 K or 298 K with adsorbate pressure ranging between 1 to 14 bar. For Cu-BDC⊃HMTA, the CO_2_ uptake recorded at 1 bar was 4.8 mmol g^−1^ (21.1 wt %), and 2.1 mmol g^−1^ (9.24 wt %) at 273 K and 298 K, respectively (Figure 6). Notably, for Cu-BDC, without the amine modification, CO_2_ uptake was measured at 1 bar as only 1.2 mmol g^−1^ (5.28 wt %), and 0.8 mmol g^−1^ (3.53 wt %) at 273 K and 298 K, respectively. At 14 bar, the CO_2_ uptake at 273 °C, and 298 °C for Cu-BDC⊃HMTA is 12 mmol g^−1^ (52.8 wt %), and 9 mmol g^−1^ (39.6 wt %), respectively (Figure 6), again markedly higher than for Cu-BDC (17.4 wt %, and 13.2 wt % at 273 K and 298 K, respectively).

### 3.7. Surface Area and Porosity of Cu-BDC⊃HMTA MOF

**Figure 7:**
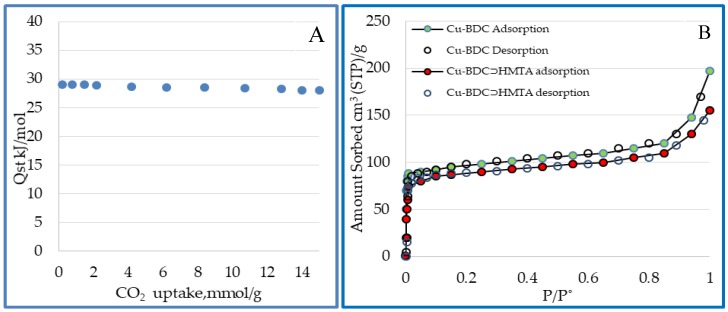
(**A**) Isosteric heats of CO_2_ adsorption onto Cu-BDC**⊃**HMTA. (**B**) N_2_ adsorption-desorption isotherms at 77 K. Adsorption is represented by hollow circles (Red = Cu-BDC**⊃**HMTA, Green = Cu BDC), and desorption is marked by closed circles.

The N_2_ adsorption isotherm for Cu-BDC⊃HMTA was recorded at 77 K (Figure 7B). The Langmuir and BET surface areas for Cu-BDC were found to be 868 m^2^/g and 708 m^2^/g, respectively, while Cu-BDC⊃HMTA revealed lower values of 683 m^2^/g (Langmuir), and 590 m^2^/g (BET) (Table 2). Although the introduction of the amine into Cu-BDC⊃HMTA reduces its surface area, the CO_2_ adsorption is increased. The presence of additional binding sites in MOFs by amine/amide incorporation has been shown to induce dispersion, and electrostatic forces that enhance CO_2_ gas adsorption (Table 2) [13]. The isosteric heat of CO_2_ adsorption (Qst) in Cu-BDC⊃HMTA was calculated from the adsorption isotherms at 273 and 298 K (Figure 7A) as 29.8 kJ mol^−1^. Such a moderate value is lower than many other MOFs (see Table 2), and is highly desirable because of the anticipated lower material regeneration energy demand. 

Qst is the heat Q released in a constant temperature calorimeter when a differential amount of gas is adsorbed at constant pressure. The Van’t Hoff isobar equation relates Qst to adsorption isotherms at different temperatures. It is derived from equating the chemical potential of the adsorbed phase, and the gas phase, applying the Gibbs Helmholtz relation, and assuming that the vapor phase behaves like an ideal gas. From experimentally obtained isotherms at a constant amount adsorbed and two different temperatures (T_1_ and T_2_), Qst is obtained by following equation:

(1) $$Qst=R\;\left(\frac{\left(lnP1-lnP2\right)}{\left(\frac{1}{T1}-\frac{1}{T2}\right)}\right)$$

where R is ideal gas constant. Isosteric heats of adsorption for Cu-BDC⊃HMTA were calculated using 273 K and 298 K isotherms using the slope of a Van’t Hoff plot against the amount adsorbed. Here, the Qst value decreases with loading, indicating strong interaction between the quadrupole moment of carbon dioxide and the adsorbent surface.

The pore size distribution obtained from BET–BJH N_2_ adsorption shows micropores at around 8.3 Å in Cu-BDC, and larger pores at 14 Å that may originate from defects or inter-crystalline gaps [14]. Unsurprisingly, a narrow pore distribution of smaller pores around 7.1 Å is observed in the Cu-BDC⊃HMTA sample.

**Figure 8:**
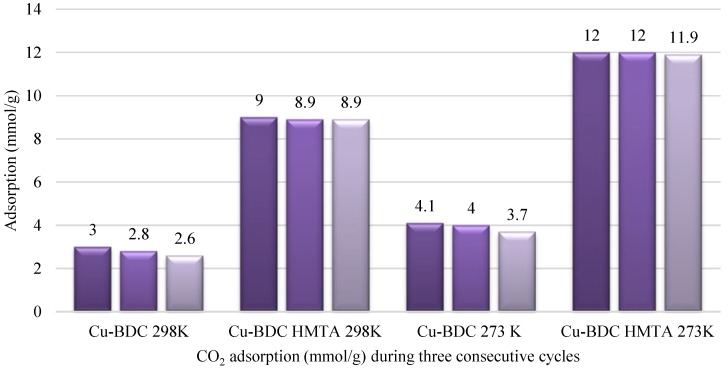
CO_2_ adsorption in mmol/g calculated at 14 bar for Cu-BDC and Cu-BDC⊃HMTA at 273 K and 298 K as shown.

The uptake of CO_2_ is only part of the utility of these materials. They also need to have reproducible uptake on more than one sorption-desorption cycle. Cu-BDC revealed a significant loss in adsorption capacity over successive operations compared to Cu-BDC⊃HMTA (Figure 8). Here, adsorption capacity calculated at 298 K and 14 bar for Cu-BDC lowered by about 14% after three cycles from 3 to 2.6 mmol/g. This decrease in adsorption capacity over successive cycles was more prominent at higher temperature compared to lower temperature (273 K) adsorption. In contrast, Cu-BDC⊃HMTA demonstrated much lower percentage decline in CO_2_ uptake over three successive adsorption cycles (1.1% at 298 K and 0.83% at 273 K). 

## 4. Conclusions

In summary, we report the simple modification of a Cu-BDC MOF during synthesis by the incorporation of a hexamethylenetetramine additive. The Cu-BDC⊃HMTA MOF material forms as a crystalline solid with rod-like crystallites. Thermogravimetric studies reveal that Cu-BDC⊃HMTA is more thermally stable than Cu-BDC MOF. Moreover, carbon dioxide adsorption studies for these samples reveal markedly better carbon dioxide uptake by the amine-modified framework (5.25 wt % for Cu-BDC, and 21.2 wt % for Cu-BDC⊃HMTA, respectively, at 273 K and 1 bar). The addition of nitrogen atoms by the incorporation of HMTA leads to the enhanced adsorption of CO_2_ gas, which we ascribe to favorable interactions [26] between CO_2_ molecules and the nitrogen-modified pores [27]. The modified MOF, Cu-BDC⊃HMTA, also displays enhanced cyclic stability and can be reused over three cycles. This study describes a cost-effective strategy for the incorporation of amine groups in MOF structures, for enhanced CO_2_ capture applications, using HMTA as a cheap additive. This serves as a low-cost alternative to expensive amine based ligands that are often custom-built to make MOFs for carbon dioxide capture. Future studies are needed to address the longer-term stability of Cu-BDC⊃HMTA and stability to contaminants.

## Figures and Tables

**Table 1:** Elemental composition of Cu-BDC and Cu-BDC⊃HMTA.

Elemental Composition	Calculated by Elemental Analyzer			Calculated by EDS			
MOF Sample	C	H	N	C	O	N	Cu
Cu-BDC	44.03 (44.07)	3.64 (3.69)	4.63 (4.67)	45.94 (44.0)	27.27 (26.62)	5.70 (4.67)	21.09 (21.13)
Cu-BDC⊃HMTA	45.80 (45.77)	4.4 (4.39)	15.30 (15.26)	47.81 (45.77)	18.59 (17.41)	16.30 (15.26)	17.30 (17.27)

Note: Theoretical values in brackets and calculated values outside brackets.

**Table 2:** Surface area, CO_2_ uptake and Qst values for selected Cu-based MOFs.

Material	BET (m^2^/g)	Temperature (K)	Pressure (bar)	CO_2_ Adsorption (wt %)	Qst (KJ mol^−1^)	Reference
Cu (TATB)	3360	293	-	-	61	[13]
[Cu_3_(TDPAT)]	1938	273	1	25.8	42.2	[14]
${\mathrm{Cu}}_{2}{\left(H_{2}O\right)}_{2}\mathrm{BDPO}$	2447	273	1	40.1	25.4	[15]
[Cu_4_(μ4-O)Cl_2_(COO)_4_N4]	2690	273	10	27.3	36.5	[16]
$\mathrm{Cu}{\left(\mathrm{pia}\right)}_{2}\left({\mathrm{SiF}}_{6}\right){\left(\mathrm{EtOH}\right)}_{2}{\left(H_{2}O\right)}_{12}$	285	296	1	5.5	30	[17]
$[{\mathrm{Cu}}_{3}{\left(\mathrm{BTB}{)}^{-6}\right]}_{n}$	3288	273	20	157	-	[18]
${\left[{\mathrm{Cu}}_{3}L_{2}(H_{2}O\right)}_{5}]$	2690	273	1	27.3	-	[19]
$\left[{\mathrm{Cu}}_{2}\mathrm{PDAI}\left(H_{2}O\right)\right]$	1372	273	1	28.6	26.3	[20]
$\left[{\mathrm{Cu}}_{2}\left(\mathrm{TCMBT}\right)\left(\mathrm{bpp}\right)\left(\mu_{3}-\mathrm{OH}\right)\right]\cdot 6H_{2}O$	808	298	20	25.5	26.7	[21]
$\left[{\mathrm{Cu}}_{2}\left({\mathrm{BDPT}}^{4-}\right){\left(H_{2}O\right)}_{2}\right]$	1400	273	1	30.7	22.5	[22]
en-CuBTTri	345	298	1	58.2	90	[23]
en@CuBTC	-	298	1	19.5	30	[24]
mmen-CuBTTri	870	298	1	15.4	-	[25]
Cu-BDC	708	273	1 (14)	5.28 (17.4)	-	Present study
Cu-BDC⊃HMTA	590	273	1 (14)	21.2 (52.8)	29.8	Present study

## References

[1] Bao Z., Yu L., Ren Q., Lu X., Deng S. (2011). Adsorption of CO_2_ and CH_4_ on a magnesium-based metal organic framework. J. Colloid Interface Sci..

[2] Liu J., Thallapally P.K., McGrail B.P., Brown D.R., Liu J. (2012). Progress in adsorption-based CO_2_ capture by metal-organic frameworks. Chem. Soc. Rev..

[3] Sumida K., Rogow D.L., Mason J.A., McDonald T.M., Bloch E.D., Herm Z.R., Bae T.-H., Long J.R. (2012). Carbon Dioxide Capture in Metal–Organic Frameworks. Chem. Rev..

[4] Easun T.L., Nevin A.N. (2019). Metal nodes and metal sites in metal–organic frameworks. Organomet. Chem..

[5] Li J.R., Sculley J., Zhou H.C. (2012). Metal–Organic Frameworks for Separations. Chem. Rev..

[6] Han D., Jiang F.L., Wu M.Y., Chen L., Chen Q.H., Hong M.C. (2011). A non-interpenetrated porous metal-organic framework with high gas-uptake capacity. Chem. Commun..

[7] Furukawa H., Cordova K.E., O’Keeffe M., Yaghi O.M. (2013). The chemistry and applications of metal-organic frameworks. Science.

[8] Furukawa H., Muller U., Yaghi O.M. (2015). “Heterogeneity within order” in metal-organic frameworks. Angew. Chem. Int. Ed..

[9] Mason J.A., McDonald T.M., Bae T.-H., Bachman J.E., Sumida K., Dutton J.J., Kaye S.S., Long J.R. (2015). Application of a high-throughput analyzer in evaluating solid adsorbents for post combustion carbon capture via multicomponent adsorption of CO_2_, N_2_, and H_2_O. J. Am. Chem. Soc..

[10] Carson C.G., Hardcastle K., Schwartz J., Liu X., Hoffmann C., Gerhardt R.A., Tannenbaum R. (2009). Synthesis and structure characterization of copper terephthalate metal-organic frameworks. Eur. J. Inorg. Chem..

[11] Hexamethylenetetraamine; Sigma-Aldrich Corporation online (UK) pricing 11th April 2019 of £62.80 for 4 kg, ReagentPlus®, 99%. https://www.sigmaaldrich.com/catalog/product/sial/h11300?lang=en&region=PK.

[12] Ilyes E., Florea M., Madalan A.M., Haiduc I., Parvulescu V.I., Andruh M. (2012). A Robust Metal–Organic Framework Constructed from Alkoxo-Bridged Binuclear Nodes and Hexamethylenetetramine Spacers: Crystal Structure and Sorption Studies. Inorg. Chem..

[13] Caskey S.R., Wong-Foy A.G., Matzger A.J. (2008). Dramatic tuning of carbon dioxide uptake via metal substitution in a coordination polymer with cylindrical pores. J. Am. Chem. Soc..

[14] Yang Y., Lin R., Ge L., Hou L., Bernhardt P., Rufford T.E., Wang S., Rudolph V., Wang Y., Zhu Z. (2015). Synthesis and characterization of three amino-functionalized metal-organic frameworks based on the 2-aminoterephthalic ligand. Dalton Trans..

[15] Hu Z., Nalaparaju A., Peng Y., Jiang J., Zhao D. (2016). Modulated hydrothermal synthesis of UIO-66(Hf) type Metal Organic Frameworks for Optimal Carbon Dioxide Separation. Inorg. Chem..

[16] Zheng B., Bai J., Duan J., Wojtas L., Zaworotko M.J. (2011). Enhanced CO_2_ Binding Affinity of a High-Uptakerht-Type Metal−Organic Framework Decorated with Acylamide Groups. J. Am. Chem. Soc..

[17] Cai J.F., Wang H.Z., Wang H.L., Duan X., Wang Z.Y., Cui Y.J., Yang Y., Chen B.L., Qian G.D. (2015). An amino-decorated NbO-type metal–organic framework for high C_2_H_2_ storage and selective CO_2_ capture. RSC Adv..

[18] Xiong S., He Y., Krishna R., Chen B., Wang Z. (2013). Metal–Organic Framework With Functional Amide Groups For Highly Selective Gas Separation. Cryst. Growth Des..

[19] Zheng B., Yang Z., Bai J., Li Y., Li S. (2012). High and selective CO_2_ capture by two mesoporous acylamide-functionalized rht-type metal–organic frameworks. Chem. Commun..

[20] Duan J., Yang Z., Bai J., Zheng B., Li Y., Li S. (2012). Highly selective CO_2_ capture of an agw-type metal–organic framework with inserted amides: Experimental and theoretical studies. Chem. Commun..

[21] Park J., Li J.R., Chen Y.P., Yu J., Yakovenko A., Wang Z.U., Sun L.B., Balbuena P.B., Zhou H.C. (2012). A Versatile Metal–Organic Framework For Carbon Dioxide Capture and Cooperative Catalysis. Chem. Commun..

[22] Lu Z., Xing H., Sun R., Bai J., Zheng B., Li Y. (2012). Water Stable Metal–Organic Framework Evolutionally Formed from a Flexible Multidentate Ligand with Acylamide Groups for Selective CO_2_ Adsorption. Cryst. Growth Des..

[23] Wang Z., Zheng B., Liu H., Lin X., Yu X., Yi P., Yun R. (2013). High Capacity Gas Storage by a Microporous Oxalamide Functionalized NbO-Type Metal–Organic Framework. Cryst. Growth Des..

[24] Demessence A., D’Alessandro D.M., Foo M.L., Long J.R. (2009). Strong CO_2_ binding in a water stable, triazolate-bridged metal-organic framework functionalized with ethylenediamine. J. Am. Chem. Soc..

[25] Montoro C., Garcia E., Calero S., Perez-Fernandez M.A., Lopez A.L., Barea E., Navarro J. (2012). Functionalization of MOF open metal sites with pendant amines for CO_2_ capture. J. Mater. Chem..

[26] McDonald T.M., D’Alessandro D.M., Krishna R., Long J.R. (2011). Enhanced carbon dioxide capture upon incorporation of N,N’-dimethylethylenediamine in the metal-organic framework CuBTTri. Chem. Sci..

[27] Karmakar A., Desai A.V., Manna B., Joarder B., Ghosh S.K. (2015). An Amide-Functionalized Dynamic Metal–Organic Framework Exhibiting Visual Colorimetric Anion Exchange and Selective Uptake of Benzene over Cyclohexane. Chem. A Eur. J..

